# Upregulated Autophagy in Sertoli Cells of Ethanol-Treated Rats Is Associated with Induction of Inducible Nitric Oxide Synthase (iNOS), Androgen Receptor Suppression and Germ Cell Apoptosis

**DOI:** 10.3390/ijms18051061

**Published:** 2017-05-15

**Authors:** Akio Horibe, Nabil Eid, Yuko Ito, Hitomi Hamaoka, Yoshihisa Tanaka, Yoichi Kondo

**Affiliations:** Department of Anatomy and Cell Biology, Division of Life Sciences, Osaka Medical College, 2-7 Daigaku-machi, Takatsuki, Osaka 569-8686, Japan; horibe@kubomizuki.or.jp (A.H.); an1006@osaka-med.ac.jp (Y.I.); an1021@osaka-med.ac.jp (H.H.); an1028@osaka-med.ac.jp (Y.T.); konchan@osaka-med.ac.jp (Y.K.)

**Keywords:** ethanol, autophagy, apoptosis, androgen receptor (AR), inducible nitric oxide synthase (iNOS), Sertoli, transcription factor EB (TFEB)

## Abstract

This study was conducted to investigate the autophagic response of Sertoli cells (SCs) to acute ethanol toxicity using in vivo and in vitro models. Adult Wistar rats were intraperitoneally injected with either 5 g/kg ethanol or phosphate-buffered saline (for the control group) and sacrificed 0, 3, 6 and 24 h after injection. Compared to the control group, enhanced germ cell apoptosis was observed in the ethanol-treated rats (ETRs) in association with upregulation of iNOS and reduced expression of androgen receptor protein levels in SCs, which were resistant to apoptosis. Meanwhile, autophagy was upregulated in ETR SCs (peaking at 24 h) compared to the control group, as evidenced by transcription factor EB (TFEB) nuclear translocation, enhanced expression of microtubule-associated protein 1 light chain3-II (LC3-II), lysosome-associated membrane protein-2 (LAMP-2), pan cathepsin protein levels and reduced expression of p62. This upregulation of SC autophagy was confirmed ultrastructurally by enhanced formation of autophagic vacuoles and by immunofluorescent double labelling of autophagosomal and lysosomal markers. Study of cultured SCs confirmed enhanced autophagic response to ethanol toxicity, which was cytoprotective based on decreased viability of SCs upon blocking autophagy with 3-methyladenine (3-MA). The results highlighted the molecular mechanisms of prosurvival autophagy in ETR SCs for the first time, and may have significant implications for male fertility.

## 1. Introduction

Testes contain unique branched Sertoli cells (SCs) that support germ cells physically, metabolically (via Warburg-like effects) and immunologically by creating an immune-privileged environment [1,2,3]. These SC prosurvival roles may extend to other cells within the testes (such as Leydig cells) or even outside the testes with transplantation or co-transplantation to diseased organs and co-culturing with other cells [4,5,6]. These protective roles of SCs for various cells are most probably based on resistance to apoptotic death signals, but the specific mechanisms of SC survival in normal and stressful environments are not clearly understood.

A pressing body of evidence indicates that autophagy represents a prosurvival pathway for clearance of damaged cellular components; specifically, this applies with various stressors such as oxidative, endoplasmic reticulum (ER) and nitrative stress, mitochondrial damage, lipid overload and androgen suppression [7,8,9,10]. Autophagy is characterized by the formation of isolation membranes, which sequester cellular components producing microtubule-associated protein 1 light chain 3 (LC3)-mediated autophagosomes. The latter then fuse with lysosomes to form autolysosomes via lysosome-associated membrane protein-2 (LAMP-2) for cargo clearance by lysosomal cathepsins [11,12,13]. Importantly, the induction of autophagy in response to various stressors such as oxidative stress is positively controlled by overexpression and nuclear translocation of the transcription factor EB (TFEB)—a master upregulator of autophagy proteins and an activator of lysosomal biogenesis [14,15,16,17,18].

A number of recent reports have highlighted autophagy’s central role in SC survival upon exposure to certain toxicants and acute physical stress. For example, in response to acute heat stress (HS), cultured boar SCs exhibited high autophagic activity, which has been found to be associated with an antiapoptotic mechanism as evidenced by enhanced apoptosis of HS-treated SCs on autophagy inhibition. This enhanced autophagic response in stressed SCs also accelerated the secretion of lactate (an essential substrate for germ cell survival) via the modulation of SLC2A3, LDHA and SLC16A1 expression levels [19]. Autophagy induction via mechanisms related to oxidative stress and mammalian target of rapamycin (mTOR) inhibition was also cytoprotective for cultured rat SCs acutely exposed to xenoestrogen 4-nonylphenol (a common environmental toxicant) [20]. Under normal conditions, autophagy upregulation in cultured rat SCs exposed to cell-derived substrates was required for phagocytosis of these substrates by SCs, indicating cooperation between the phagocytosis and autophagy mechanisms [21]. In addition, a recent study reported that autophagy degrades androgen-binding protein in rat SCs and that this autophagic clearance is negatively regulated by testosterone, which inhibits autophagy [22]. In consideration of these observations, it can be inferred that autophagy plays prosurvival roles for SCs under basal and pathological conditions. These roles may be essential for spermatogenesis and male fertility based on the various functions of SCs [4,5,6].

Alcohol (ethanol) is one of the most commonly abused drugs in the world, and most body organs are affected by its toxicity. Excessive ethanol consumption in humans and experimental animals has been reported to enhance germ cell apoptosis, inducing infertility problems via mechanisms related to oxidative stress, upregulation of inducible nitric oxide synthase (iNOS) and inflammatory cytokines, DNA damage, androgen suppression and mitochondrial dysfunction [8,23,24,25,26,27,28,29]. However, in the studies that highlighted these effects, SCs were resistant to apoptosis. The possible involvement of cytoprotective autophagy in SC resistance to acute ethanol toxicity, the molecular mechanisms regulating such autophagic response and the relationships with iNOS and androgen receptor (AR), which mediate ethanol toxicity, have not yet been reported. In the study reported here, a single dose of ethanol administered to adult rats was found to enhance germ cell apoptosis but upregulate autophagy proteins LC3, pan cathepsin, LAMP-2 and TFEB in SCs, which was associated with iNOS induction and AR suppression. This enhanced autophagic response in ethanol-treated rat (ETR) SCs appears to be a prosurvival mechanism, as observed in cultured SCs exposed to ethanol.

## 2. Results

### 2.1. Ethanol Enhanced Apoptotic Germ Cell Death, but SCs Were Resistant to Apoptosis

**Figure 1:**
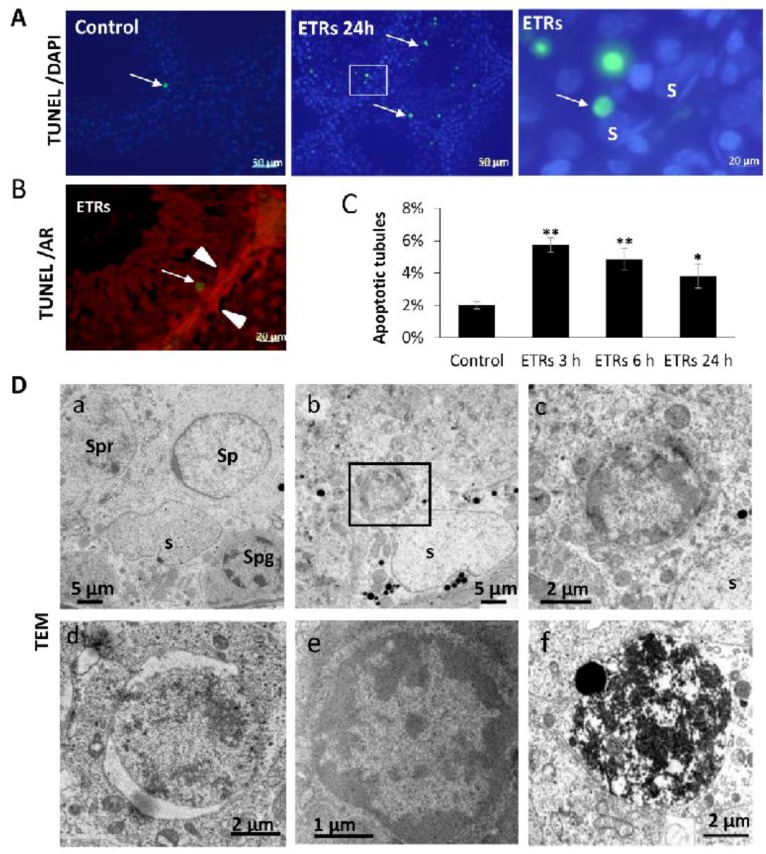
Ethanol-enhanced germ cell apoptosis. (**A**) Terminal deoxynucleotidyl transferase dUTP-mediated nick-end labelling (TUNEL) labelling of apoptotic germ cells (green reaction, white arrows). 4′,6-diamidino-2-phenylindole (DAPI) (blue staining) is used for nuclear counterstaining; (**B**) TUNEL/AR (SC marker) double labelling. The arrow heads mark nuclei of SCs expressing AR (red labelling); (**C**) Histogram showing significant increase of the percentage of apoptotic STs in ETRs. * *p* < 0.05; ** *p* < 0.01; (**D**) TEM demonstrating normal germ cells in control testis (**a**) and apoptotic germ cells in ETRs (**b**–**f**). The framed area in **b** is magnified in **c**. S: SC nucleus; Spg: spermatogonia; Sp: spermatid; Spr: spermatocyte; AR: androgen receptor; SCs: Sertoli cells; STs: seminiferous tubules; ETRs: ethanol-treated rats; TEM: transmission electron microscopy. Scale bars in A, B: 50 μm for first two panels; 20 μm for next two panels.

Adult Wistar rats were administered a single intraperitoneal injection of ethanol (5 g/kg) or phosphate buffer saline (for the control group) and sacrificed 0, 3, 6 and 24 h after injection (a model of acute alcohol toxicity) [12,23,29]. As shown in Figure 1, while SCs were mostly normal, there was a significant elevation of apoptosis in all types of germ cells in ETRs based on the terminal deoxynucleotidyl transferase dUTP-mediated nick-end labelling (TUNEL) method (A, C), TUNEL/AR (SC nuclear marker) double labelling (B) and transmission electron microscopy (TEM) (D). This enhanced germ cell apoptosis in ETRs testes was associated with germ cell detachment and the formation of giant cells, reflecting ethanol toxicity [23] (data not shown). This increase in germ cell apoptosis subsided over time (maximum at 3 h; reduced at 6 and 24 h after ethanol administration), possibly as a result of time-dependent increase in phagocytosis by SCs as reported by others in animal models of acute butylparaben toxicity [30] and the authors’ recent publications on rat hepatocytes using the same animal model of acute alcoholism [31]. TEM (Figure 1D(a–f)) and in comparison to control germ cells (a), showed the typical features of germ cell apoptosis in ETRs as chromatin condensation (b,c,e), nuclear pyknosis (d), and late degradation within SCs after phagocytosis (f), in keeping with earlier reports [23,32]. As germ cell apoptosis may be induced by oxidative-nitrosative stress in SCs and/or androgen suppression [23,24,25,26,27], the authors investigated the expression of iNOS and AR protein levels. The 24-h time point was chosen for analysis in subsequent experiments because it showed the highest expression of iNOS and autophagy proteins in the testes of ETRs as reported below, in keeping with a recent study [12].

### 2.2. Induction of iNOS and Suppression of AR Protein Levels in SCs and Interstitial Cells of ETRs

**Figure 2:**
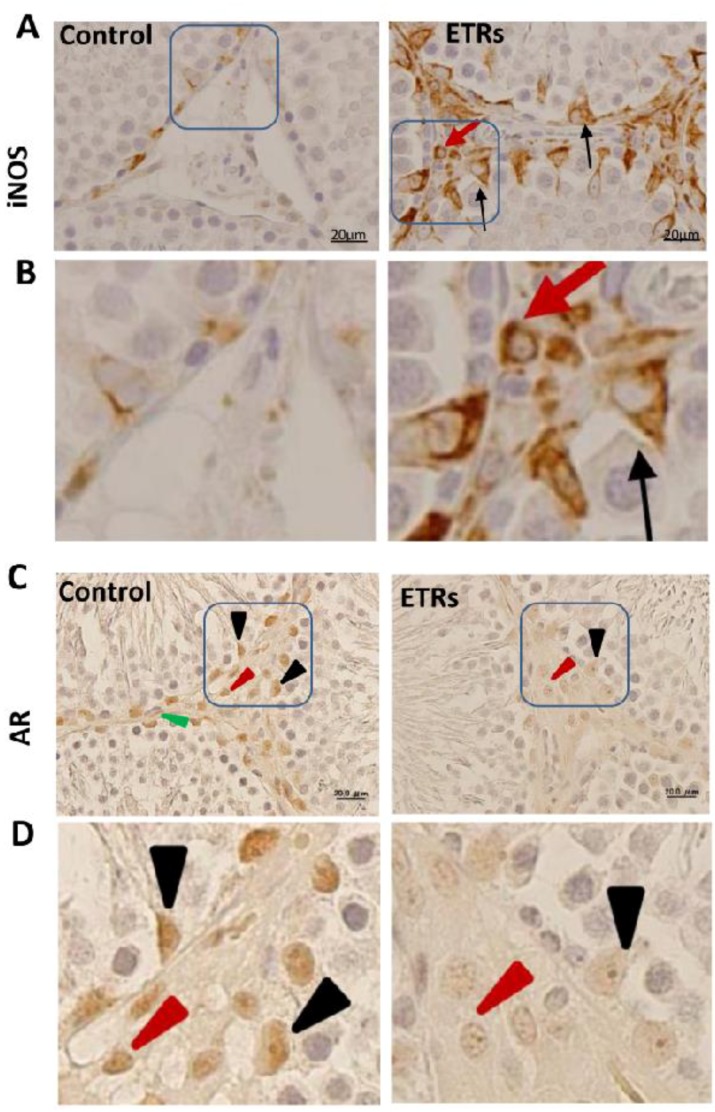
Upregulation of inducible nitric oxide synthase (iNOS) and suppression of ARs in SCs and interstitial cells of ETRs. (**A**,**B**) show the immunohistochemistry (IHC) of iNOS, while (**C**,**D**) demonstrate the IHC of AR. The framed areas in (**A**,**C**) are magnified in (**B**,**D**). The black arrows in (**A**,**B**) mark iNOS expression in SCs, and the red arrow shows its expression in an interstitial cell. Black, red and green arrow heads in (**C**,**D**) indicate nuclear expression of AR in SCs, Leydig and myoid cells, respectively. Scale bars in A, C: 20 μm.

Compared to control testis tissue with low levels of iNOS in SCs and Leydig cells, enhanced expression of this protein was observed in ETR SCs and interstitial cells (Leydig cells and macrophages) (Figure 2A,B). These observations based on immunohistochemistry (IHC) were confirmed by western blot using whole testicular tissue homogenate (Figure S1A). The upregulation of iNOS in ETR testes in the present study may be related to increased blood endotoxin levels and cytokines production by immunocytes, which are mediated by ethanol toxicity as reported by various sources, and could be responsible for induction of germ cell apoptosis via the production of excessive NO [12,23,24,25,26,33,34]. AR expression in control testis tissue (Figure 2C,D) was observed in SCs, Leydig cells and myoid cells in keeping with the outcomes of other studies [35,36,37]. However, and as a novel finding, AR expression was markedly reduced in the testes of ETRs as shown by IHC and confirmed by western blot (Figure S1B). This is in line with earlier studies reporting AR suppression in rat hepatocytes and skeletal muscles under conditions of chronic ethanol consumption [38,39]. The resistance of ETR SCs to apoptotic cell death, their expression of excessive iNOS, and the suppression of ARs may induce the activation of autophagic programming to survive the inflammatory and cytotoxic environments produced by ethanol and various stressors and toxicants, as mentioned in the introduction. Accordingly, the authors investigated autophagy mechanisms in ETR SCs.

### 2.3. Upregulated Autophagic Response in ETR SCs: Light and TEM Observations

**Figure 3:**
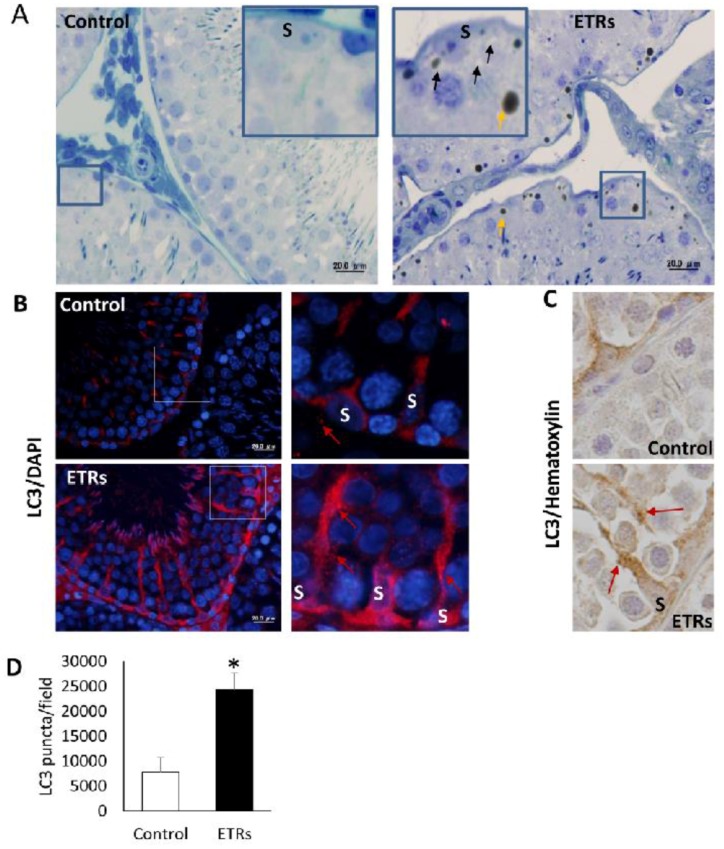
Induction and upregulation of the autophagy marker microtubule-associated protein 1 light chain 3 (LC3) in ETR SCs. (**A**) toluidine blue staining of semi-thin sections demonstrating perinuclear vacuole formation (**black arrows**) and lipid droplet (**yellow arrows**) accumulation in ETR SCs. The framed areas are magnified in the insets; (**B**,**C**) show immunofluorescence (IF) and IHC of LC3 expression, respectively. S: SC nucleus; (**D**) shows quantification of LC3 puncta. The boxed areas in B are magnified on the right. The red arrows mark LC3 puncta in SCs. * *p* < 0.05. The scale bar for left panels in B is 20 μm.

**Figure 4:**
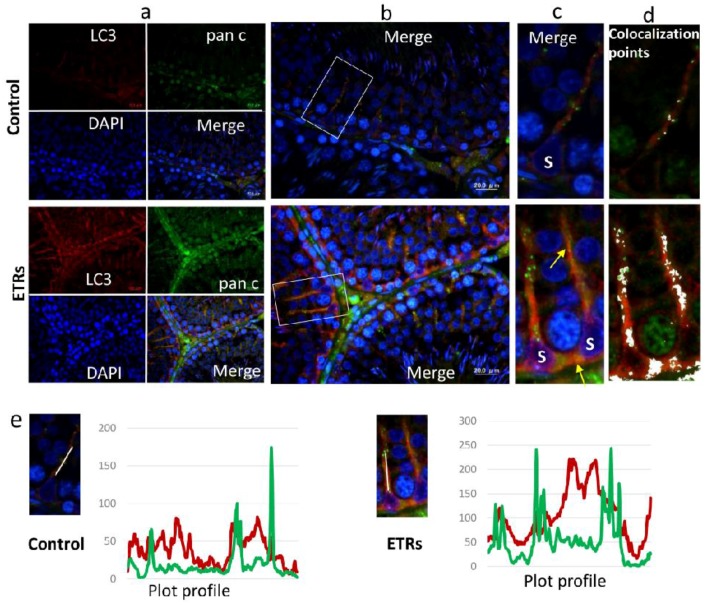
Elevated colocalization of LC3 with lysosomal cathepsins in ETR SCs, indicating enhanced autophagic activity. IF double labelling of LC3 (**red**) and pan cathepsin (pan c) (**green**) in control and ETR testes (**a**). The colocalization signals observed upon merging (**yellow-orange**) (**b**) are marked by yellow arrows (**c**) and shown as white spots (**d**) using Image J. S: SC nucleus. The histograms (**e**) are plot profiles indicating the overexpression and colocalization of LC3 (**red**) and pan cathepsin (**green**) in SCs, and correlate to the white lines shown in the images. The scale bar in **b** is 20 μm. The panels in **a** and **c**–**e** are lower and higher magnifications of **a**, respectively using image J.

**Figure 5:**
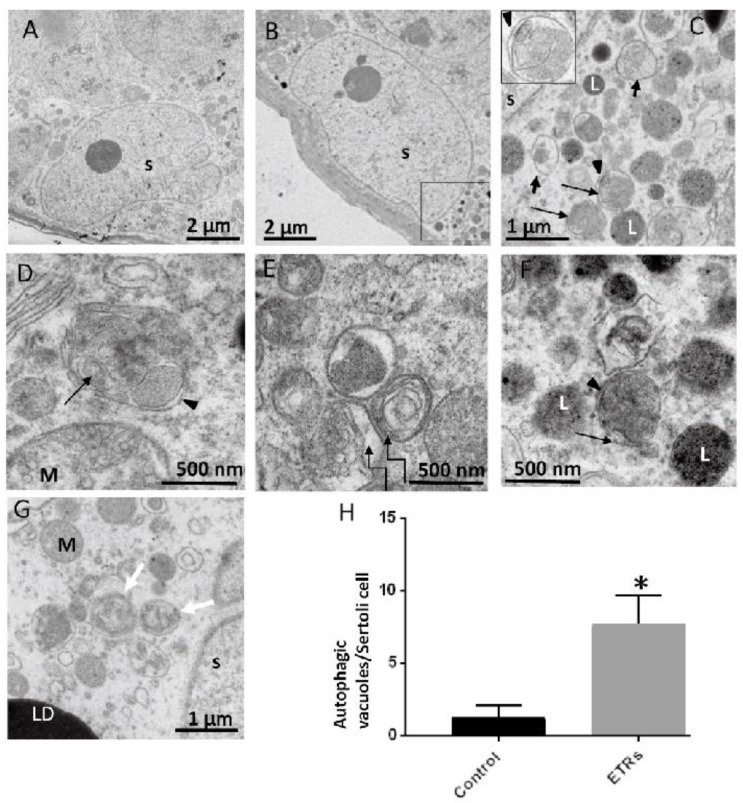
Ultrastructural features of upregulated autophagy in ETR SCs. TEM of control (**A**) and ETRs (**B**–**G**). The histogram (**H**) shows a significant increase in the number of autophagic vacuoles (AVs) in ETR SCs. The long black arrows mark autophagosomes with a double limiting membrane (**arrow heads**) (magnified in the inset in (**C**). The short arrows indicate autolysosomes. The broken arrows in (**E**) show multilamellar bodies, while the white arrows in (**G**) show autophagosomes containing fragmented mitochondria. S: SC nucleus; L: lysosome; M: mitochondria; LD: lipid droplet. * *p* < 0.05.

Toluidine blue-stained semi-thin sections from epoxy embedded blocks showed normal morphology in the testes of the control group. Meanwhile, increased testicular lipid droplet accumulation and vacuolization were observed in ETRs, and specifically in the perinuclear areas of SCs (Figure 3A). This observation of perinuclear vacuolization in SCs may reflect enhanced autophagic activity [40]. Immunofluorescence (IF) and IHC demonstrated a significant increase of LC3 puncta in ETR SCs compared to the control (Figure 3B–D), indicating enhanced LC3-II-mediated autophagosome formation [12,16,26,41]. In fact, increased LC3 expression was also observed in elongated spermatids, residual bodies and interstitial cells of ETRs, but this was not as remarkable or extensive as that observed in the case of SCs (data not shown). Furthermore, and as shown in Figure S2, Western blot results confirmed the upregulation of both forms of LC3 (LC3-I, LC3-II), supporting the findings of light-microscope observation (Figure S2A). In addition, this enhanced LC3-related autophagic activity in ETR SCs was supported by downregulation of p62 (LC3 adaptor protein and substrate) based on western blot (Figure S2B) and increased colocalization of LC3 with p62, indicating the maturation of autophagosomes (data not shown) [16,41,42]. Importantly, double-labelling of LC3 and pan cathepsin (a lysosomal marker) (Figure 4A) highlighted marked overexpression and colocalization signals in ETR SCs, indicating the formation of autolysosomes and acceleration of lysosome-related cargo degradation [11,12,31]. This finding was confirmed by immunoelectron microscopy (data not shown). TEM, the gold standard and confirmatory method for autophagy detection, [12,31,43] showed a significant increase in the formation of autophagic vacuoles (autophagosomes and autolysosomes) in ETR SCs (Figure 5A–H). Compared to control SCs (Figure 5A), enhanced formation is observed for double-membrane autophagosomes and single-membrane autolysosomes (Figure 5B–D). In addition, multilamellar bodies sequestrating the rough endoplasmic reticulum (ER) (Figure 5E) and autophagosomes containing damaged mitochondria (Figure 5F,G) were observed in ETR SCs, indicating the enhanced degradation of organelles [11,12,31,43].

### 2.4. Ethanol-Induced Autophagy in SCs Is Associated with Upregulation and Nuclear Translocation of TFEB

**Figure 6:**
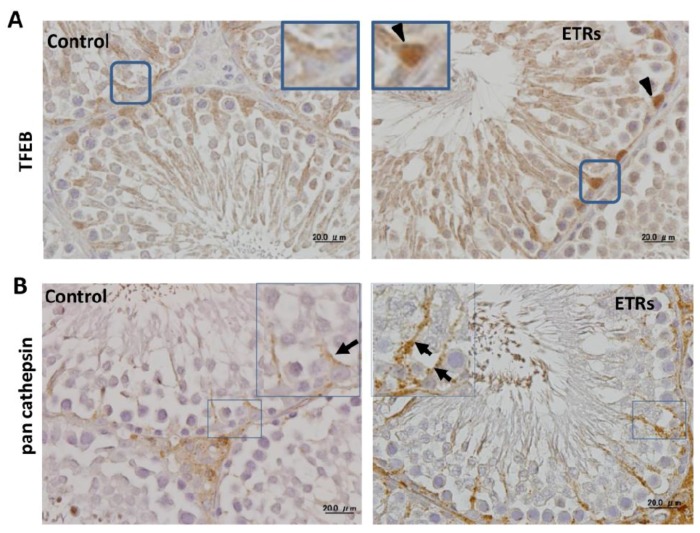
Upregulation and transcription factor EB (TFEB) nuclear translocation in ETR SCs are associated with increased expression of lysosomal proteins. (**A**,**B**) show IHC of TFEB and pan cathepsin, respectively. The framed SC nuclei in (**A**) are magnified in the insets, and arrow heads show nuclear expression of TFEB. The framed areas of SCs in (**B**) are magnified in the insets. Black arrows show pan cathepsin expression in SCs.

A novel finding of the current study was the overexpression and nuclear translocation of TFEB in ETR SCs (Figure 6A). As shown in control testis tissue, the expression of TFEB was mainly cytosolic, but increased expression and nuclear translocation of TFEB were observed in ETR SCs (Figure 6A, inset) and confirmed by immunoelectron microscopy (Figure S3). These immunohistochemical and immunoelectron microscopic findings were supported by western blotting of whole testes lysate (Figure S4A). This is in line with reports indicating the essential role of TFEB in stress-mediated upregulation of autophagy lysosomal proteins and reformation of lysosomes in various tissues [14,15,16,17,18]. This possibility of TFEB-mediated elevation of autophagy in ETR SCs was supported by upregulation of LAMP-2 (Figure S4B) and pan cathepsin (Figure 6B). TFEB upregulation appears specific; in the authors’ investigation regarding the expression of another transcription factor for autophagy (Foxo3a; [43]), no differences between control and ETR testes were found (data not shown).

### 2.5. Ethanol-Induced Autophagy in Cultured SCs as a Prosurvival Mechanism

**Figure 7:**
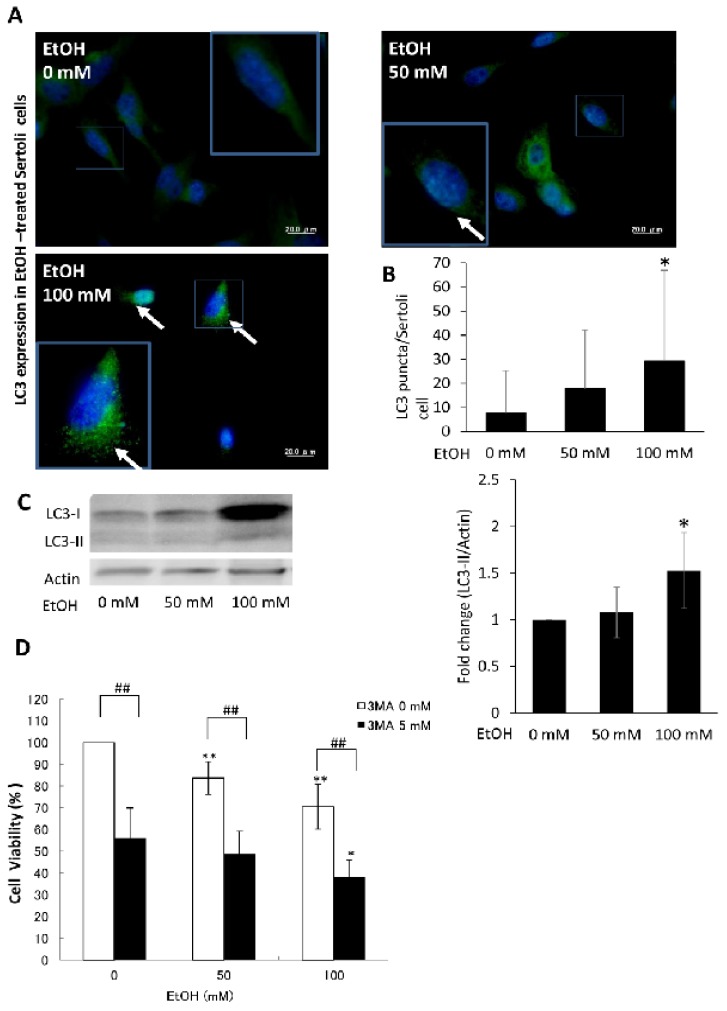
Ethanol (EtOH) enhanced autophagy in cultured SCs as an apparent prosurvival mechanism. (**A**,**B**) show LC3 staining and quantification, and (**C**) shows western blot of LC3-II. The framed areas in A are magnified in the insets. The white arrows indicate LC3 puncta; (**D**) shows a histogram indicating reduced viability of SCs on autophagy blocking with 3-methyladenine (3-MA). * *p* < 0.05; ** & ## *p* < 0.01 significantly different from the corresponding group. The statistical analysis of western blot is based on five independent experiments.

To provide direct evidence for the cytoprotective role of autophagy in ETR SCs, cultured TM4 SCs were exposed to various concentrations of ethanol for 24 h. As shown in Figure 7A, compared to untreated cells, administration of ethanol at concentrations of 50 and 100 mM enhanced formation of LC3 puncta, which was significant at the higher dose (Figure 7B). This was confirmed by upregulation of LC3-II using western blot, which was statistically significant (Figure 7C) and downregulation of p62 in ethanol-exposed SCs compared to the control (Figure S5). Blocking autophagy with 3-methyladenine (3-MA), an inhibitor of early autophagosome formation [44,45], resulted in a marked reduction of cell viability in cultured ethanol-treated SCs (Figure 7D), indicating the prosurvival role of autophagy as supported by other studies [19,20,34,41,44,45].

## 3. Discussion

Relatively few studies have investigated the roles of autophagy in ethanol-induced testicular damage as compared to studies on other organs such as the liver and brain, despite the important roles of autophagy in counteracting various toxic insults that may be critical to male fertility [7,8,9,46]. The novel finding of the current study relates to enhanced autophagic response in ETR SCs—a response that may be mediated by TFEB and related to (1) increased germ cell apoptosis; (2) upregulation of iNOS; and (3) AR suppression. This upregulated autophagy in SCs is a prosurvival mechanism that may be essential for mitigating acute ethanol-induced testicular damage and promoting recovery.

The elevated germ cell apoptosis observed in ETRs in the current study is consistent with the apoptotic effect of ethanol seen in various organs with the same animal model [12,29,31,34,44]. This ethanol-induced germ cell apoptosis may be mediated by mitochondrial and death receptor pathways via mechanisms related to oxidative stress, induction of iNOS, androgen suppression and others, as the authors reported previously in relation to animal models of chronic alcoholism [11,23,25,28]. The enhanced expression of iNOS observed in ETR SCs in the current study may be related to increased presence of cytokine and inflammatory mediators [12,23,24,25,26,29,33,34]. However, the increased expression of iNOS observed in ETR interstitial cells in the current study (most probably by macrophages) may suppress androgen production [47,48,49], which may be an additional factor in enhanced germ cell apoptosis and detachment. In addition, suppression of androgens may be a mechanism of AR downregulation in various somatic cells of the ETRs observed in the current study. However, the presence of other mechanisms for AR suppression by ethanol cannot be ruled out. Further studies are needed to explore these mechanisms in light of AR’s essential role in orchestrating the protective functions of SCs for germ cells [35,36,37].

The current study showed enhanced autophagy in ETR SCs based on the upregulation of autophagy proteins, LC3, LAMP-2, pan cathepsin and downregulation of p62. This enhanced autophagy in ETR SCs may be induced by excessive iNOS production as a mechanism to dampen its production, subsequently reducing the inflammatory response [12,34]. In addition, AR suppression in ETR SCs may be an additional stimulus for enhanced autophagy [10,50].

The overexpression of autophagy genes in ETR SCs was associated with upregulation and nuclear translocation of TFEB, indicating that the enhanced autophagic response may be regulated by this factor as reported in relation to hepatocytes of acute ETRs [16]. TFEB nuclear translocation has reportedly been induced by various stress signals activating autophagy, such as oxidative stress. TFEB nuclear translocation is also essential for lysosomal formation; this may account for enhanced expression of the lysosomal proteins LAMP-2 and pan cathepsin in ETR SCs [14,15,16,17,18,34]. Double IF labelling of LC3 and pan cathepsin in ETR SCs showed enhanced colocalization indicating increased autophagic activity and possibly enhanced heterophagy of apoptotic germ cells via LC3-associated phagocytosis [12,21,31,51,52,53]. TEM in the current study demonstrated the increased formation of autophagic vacuoles in ETR SCs, which appeared viable, confirming the results of light-microscope observation. The presence of fragmented organelles such as mitochondria was also observed in these vacuoles; this may be an antiapoptotic mechanism, as damaged mitochondria may release proapoptotic factors as caspases that could be lethal [9,31]. The question of whether there is a specific mechanism for mitochondrial degradation via autophagy in ETR SCs (such as a PINK1-Parkin mitophagy pathway) is under investigation [31,43].

The upregulated autophagy observed in ETR SCs appears to be a prosurvival mechanism, as no apoptotic SCs were detected using the TUNEL method, TUNEL/AR double labeling and TEM. This cytoprotective role for autophagy has also been observed in thymic macrophages [12], hepatocytes [31,44] and astrocytes [34] with the same animal model of acute ethanol toxicity. To provide direct proof for the prosurvival role of autophagy in SCs, the authors exposed cultured SCs to various concentrations of ethanol and found that blocking of autophagy by 3-MA resulted in reduction of viability, as also observed in similar studies of cultured SCs with various models of acute stress [19,20] and in cultured hepatocytes exposed to ethanol [44]. Blocking autophagy with 3-MA mitigated viability both in ethanol-exposed SCs and in untreated cells, indicating that autophagy is essential for the survival of SCs under normal and ethanol-exposed conditions [44].

**Figure 8:**
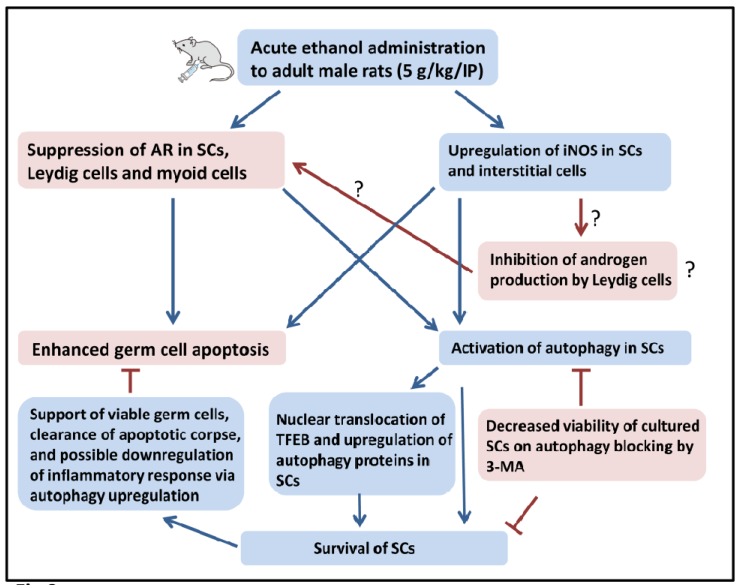
A schema showing the molecular mechanisms, evidences and consequences of ethanol-induced upregulation of autophagy in SCs.

Notably, the upregulation of SC autophagy in ETRs observed in the current study may also be cytoprotective for germ cells, as it has been reported that enhanced autophagy in HS-treated SCs is essential for the supply of lactate—an essential antiapoptotic substrate for germ cell survival—via Warburg-like mechanisms [2,3,19]. Moreover, this enhanced autophagic response in SCs may be required for phagocytosis of apoptotic germ cells [12,21]. Further studies are needed to explore Warburg mechanisms and autophagy-related phagocytosis in SCs in animal models of ethanol toxicity. A schema showing a summary of the results and analysis of the current study is provided in Figure 8.

In brief, this study helped to clarify the molecular pathology of acute ethanol-induced testicular damage and enhanced autophagy in ETR SCs, which is a prosurvival mechanism related to ethanol toxicity (germ cell apoptosis, iNOS upregulation and AR suppression). This enhanced autophagic response in SCs may be essential for spermatogenesis, and may have significant clinical implications for male fertility via the manipulation of autophagy proteins and TFEB by specific pharmacological and natural products.

## 4. Materials and Methods

### 4.1. Study Approval

The animals used in this study were maintained and treated in compliance with the guidelines set by the Experimental Animal Research Committee of Osaka Medical College (Approved by Animal Research Committee of Osaka Medical College on 10/28/2013, under code, 25090).

### 4.2. Antibodies and Kits

The antibodies and kits used are listed in Table 1, Table 2 and Table 3.

### 4.3. Animal Experiment

Twelve-week-old adult male Wistar rats (250–300 g) were purchased from SLC Japan (Shizuoka, Japan) and given a single intraperitoneal injection of 40% ethanol (5 g/kg), which is consistent with animal models of acute (binge) ethanol administration [12,13,54,55,56,57]. A control group was also treated with phosphate buffered saline. For time-course studies, animals are sacrificed and testicular samples are taken at 0, 3, 6 and 24 h after ethanol administration (thee animals at each time point). The time of 24 h was selected for this study on autophagy in SCs because it is the time of highest autophagic activity (as outlined in the results section). The testes were divided into small pieces and fixed in either 4% paraformaldehyde for light-microscope observation and production of paraffin blocks, or 2% paraformaldehyde and 2.5% glutaraldehyde in 0.1 M phosphate buffer for TEM and embedding in epoxy resin [12,31]. Unfixed testicular pieces were frozen in liquid nitrogen for western blot analysis.

### 4.4. Cell Culture and Treatment

A TM4 (#88111401, ECACC, Salisbury, Wilts, UK) SC line was cultured in a Dulbecco’s Modified Eagle’s medium (DMEM)/F-12 nutrient mixture (D8437, Sigma-Aldrich, St. Louis, MO, USA) containing 5% horse serum (MIC2921149, MP Bio, Tokyo, Japan) and 2.5% fetal bovine serum (FBS) (S1820, Bio West, Nuaille, France) at 37 °C in 5% CO_2_. The cells were treated with ethanol (50 or 100 mmol/L,) with and without autophagy inhibitor 3-MA (sc-205596, Santa-Cruz Biotechnology, TX, USA) as reported by others [41,44].

### 4.5. Blocking Autophagy in Cultured SCs with 3-MA and Cell Viability Assay

Cell viability was assessed using CellTiter-Blue^®^ Cell Viability assay (G8080, Promega, Madison, WI, USA). Cells were plated at a density of 2–3 × 10^4^ cells per well in a 96-well plate (Thermo Fisher Scientific, Waltham, MA, USA) and incubated overnight, and were then pre-treated with 5 mM 3-MA for 1 h before being exposed to ethanol for 24 h. After incubation, 20 µL of CellTiter-Blue reagent was added to each, followed by a further hour of incubation. Absorbance (Ex544/Em590 nm) was measured using a Fluoroskan Ascent™ Microplate Fluorometer (Thermo Fisher Scientific, Waltham, MA, USA) as recently reported by the authors and others [44,45,58].

### 4.6. Histopathological Analysis

Paraffin block sections (thickness: 4 μm) stained with hematoxylin and eosin (H&E) and toluidine blue-stained semi-thin sections from epon-embedded tissues were observed using a light microscope for histopathological analysis and selection of the area of interest for TEM [12,31].

### 4.7. TUNEL Assay and TUNEL/AR Double Labelling

TUNEL was performed in line with manufacturer protocols as previously reported [12,31,58]. The percentage of apoptotic seminiferous tubules (STs; tubules containing three or more apoptotic germ cells based on their cross sections) was calculated as previously reported [23]. 4′,6-diamidino-2-phenylindole (DAPI) was used for nuclear counterstaining, and the sections were observed under an immunofluorescence microscope (BX41, Olympus, Tokyo, Japan). TUNEL/AR was performed in two steps [31]. First, TUNEL labelling was conducted, and then (after washing in a buffer), IF staining was applied for AR using the specific primary and secondary antibodies shown in Table 1, Table 2 and Table 3.

### 4.8. Immunohistochemistry

The 4-μm-thick sections were deparaffinized and underwent a process of antigen retrieval, blocking of endogenous peroxidase activity, washing in PBS, blocking of nonspecific binding of antibodies, and incubation for 1 h at room temperature with primary antibodies for iNOS, AR, LC3, TFEB and pan cathepsin (Table 1). The sections were then incubated with Envision+ Single or Dual link (DAKO, Kyoto, Japan) for 30 min at room temperature [12,23,31,58] and observed under the Olympus BX41microscope (BX41, Olympus, Tokyo, Japan).

### 4.9. Immunofluorescence Single and Double Labelling

The 4-μm-thick sections were deparaffinized and underwent a process of rehydration, blocking of nonspecific antibody binding and incubation for 2 h at room temperature with the specific primary antibody LC3 (Table 1) for single labelling or LC3 with pan cathepsin for double labelling. The sections were then incubated for a further 30 min at room temperature with Alexa Fluor 594 conjugated secondary antibody for single labeling. Alexa Fluor 594 and VectaFluor™ R.T.U. DyLight^®^ 488 was used for LC3/pan cathepsin double labelling (sequential method), and DAPI was used for nuclear counterstaining as we reported [11,19,31,43]. The sections were observed under the Olympus BX41 immunofluorescence microscope. Quantification of LC3 puncta/field in SCs was performed using Image J (available online: http://imagej.nih.gov/ij/) as reported previously [11,12,23,58]. The colocalization areas of LC3/pan cathepsin were marked using the program’s image highlighter function. For culture study, IF staining of LC3 in SCs was performed after fixation in 4% paraformaldehyde and permeabilization using Triton as recently reported by the authors [58]. Quantification of LC3 puncta in SCs was performed as also reported recently by the authors [31].

### 4.10. Line Profile Plots for Co-Localization Analysis

Line profiles from the two fluorescent channels were analyzed using Image J software as reported previously by the authors and others [31,59]. Line profiles reflect intensity and colocalization of two different proteins as overlapping red and green peaks (the vertical axis shows intensity of fluorescence, and the horizontal axis indicates distance).

### 4.11. TEM and Quantitative Analysis

Ultra-thin sections (70 nm) from blocks embedded in epoxy resin were cut out with a diamond knife, stained first with Uranyless stain (Delta Microscopy, Mauressac, France) [60,61] and then with lead citrate, and examined using an H-7650 transmission electron microscope (Hitachi, Japan). For quantification of autophagic vacuoles in SCs, 10 lower-magnification photomicrographs from the testes of controls and ETRs (×2500–3000) were selected (each image containing at least one SC nucleus) [31,62,63].

### 4.12. Western Blot Analysis

Whole testicular tissues were homogenized in a modulated RIPA buffer followed by centrifugation at 12,000× *g* for 10 min at 4 °C and collection of the supernatant, which was then mixed with a loading buffer and boiled for 5 min. The supernatant was electrophoresed on 12% sodium dodecyl sulfate polyacrylamide gel and transferred onto a polyvinylidene difluoride membrane. Proteins were detected with the specific primary antibodies shown in Table 1, and then with specific peroxidase-labelled secondary antibodies as previously reported [12,58]. The relative intensity of expression of various proteins against actin (loading control) was normalized and densitometrically measured using Image J. In case of LC3, we used the LC3-II form against actin. The histograms next to the blots indicate the fold changes in protein expression in ETRs over control. This protocol of western blot and analysis was also applied to cultured SCs.

### 4.13. Statistical Analysis

Statistical analysis was performed using the PRISM program (GraphPad Software 7, San Diego, CA, USA; available online: http://www.graphpad.com/scientific-software/prism/) [31]. Differences between two or more groups were tested via analysis of variance (ANOVA), with *p* < 0.05 considered statistically significant. Student’s *t*-test was used for comparison between groups.

## 5. Conclusions

Ethanol-induced upregulation of cytoprotective autophagy in SCs may be mediated by oxidative-nitrative stress and AR suppression, and may be essential for maintenance of spermatogenesis and testicular homeostasis.

## Figures and Tables

**Table 1:** Antibodies used in current study. IHC: immunohistochemistry; IF: immunofluorescence; WB: western blot; LAMP2: lysosome-associated membrane protein-2.

Primary Antibody	Company	Catalog Number	Host	Method	Dilution
LC3	MBL	PM036	Rabbit	IHC	1:5000
				IF	1:250
				WB	1:1000
p62/SQSTM1	Novus	H00008878-M01	Mouse	IF	1:100
				WB	1:500
pan cathepsin	Santa Cruz Biotechnology	sc-6499	Goat	IHC	1:100
iNOS	abcam	ab15326	Rabbit	IHC	1:2
				IF	1:2
iNOS	abcam	ab15323	Rabbit	WB	1:150
LAMP2	Santa Cruz Biotechnology	sc-5571	Rabbit	WB	1:250
AR	Santa Cruz Biotechnology	sc-816	Rabbit	IHC	1:250
				IF	1:250
				WB	1:250
TFEB	MyBioSource	MBS9125929	Rabbit	IHC	1:2000
				IF	1:200
				WB	1:1000
FOXO3a	Cell signaling	12829	Rabbit	IHC	1:1000
				IF	1:400
				WB	1:1000
Actin	Santa Cruz Biotechnology	sc-1616	Goat	WB	1:1000

**Table 2:** Secondary antibodies and kits. IHC: immunohistochemistry; IF: immunofluorescence; WB: western blot; IEM: immunoelectron microscopy.

Secondary Antibody	Company	Catalog Number	Host	Method	Dilution
AlexaFluor 488	Thermofisher	A11001 (anti-Mouse)	Goat	IF	1:250
AlexaFluor 594	Thermofisher	A11012 (anti-Rabbit)	Goat	IF	1:250
EnVision + Dual link	DAKO	K4063		IHC	1:1
EnVision+ Single 15 nm gold-conjugated	DAKO Aurion	K4002 815.011	Goat	IHC IEM	1:1 1:25
Goat immunoglobulins	DAKO	E0466	Rabbit	IHC	1:250
Rabbit immunoglobulins	DAKO	E0432	Goat	IHC	1:250
donkey anti-goat IgG-HRP	Santa Cruz Biotechnology	SC-2304	Donkey	WB	1:10,000
donkey anti-mouse IgG-HRP	Santa Cruz Biotechnology	SC-2096	Donkey	WB	1:10,000
donkey anti-rabbit IgG-HRP	Santa Cruz Biotechnology	SC-2077	Donkey	WB	1:10,000

**Table 3:** Kits used for IHC, IF and apoptosis detection.

Name of Kit	Company	Catalog Number
Vectastain ABC Standard Kit	Vector	PK-4000
ImmPACT DAB	Vector	SK-4105
In Situ Cell Death (TUNEL) Detection Kit	Roche Diagnotics	11684817910
Western Lightning ECL Pro	PerkinElmer	NEL120001EA
VectaFluor™ R.T.U. DyLight^®^ 488	Vector	DI-3788

## Supplementary Materials

Supplementary materials can be found at http://www.mdpi.com/1422-0067/18/5/1061/s1.
