# Supplementary material for: Upregulated Autophagy in Sertoli Cells of Ethanol-Treated Rats Is Associated with Induction of Inducible Nitric Oxide Synthase (iNOS), Androgen Receptor Suppression and Germ Cell Apoptosis
Source: Int J Mol Sci. 2017 May 15;18(5):1061. doi: 10.3390/ijms18051061 (PMC5454973; doi:10.3390/ijms18051061)
Supplement: Supplementary file 1 [file ijms-18-01061-s001.pdf]

Upregulated Autophagy in Sertoli Cells of Ethanol-Treated Rats Is Associated with Induction of Inducible Nitric Oxide Synthase (iNOS), Androgen Receptor Suppression and Germ Cell Apoptosis

Akio Horibe, Nabil Eid, Yuko Ito, Hitomi Hamaoka, Yoshihisa Tanaka, and Yoichi Kondo

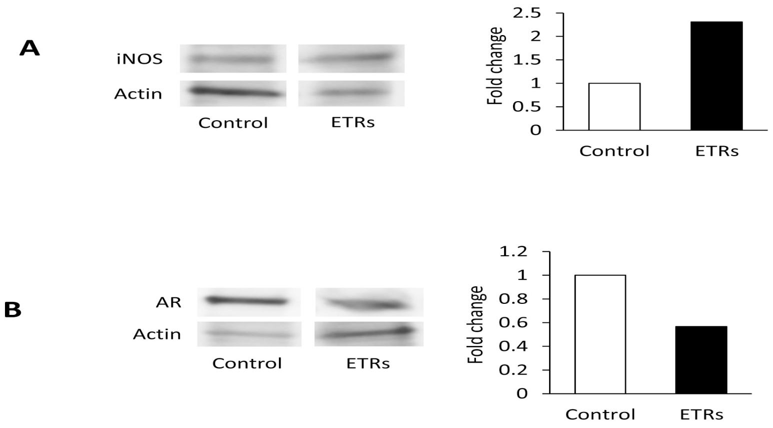

Supplementary figure 1

Figure S1. Western blotting of whole testicular tissue homogenate, showing upregulation of iNOS (A) and downregulation of AR (B) in ETRs, confirming immunohistochemical study.

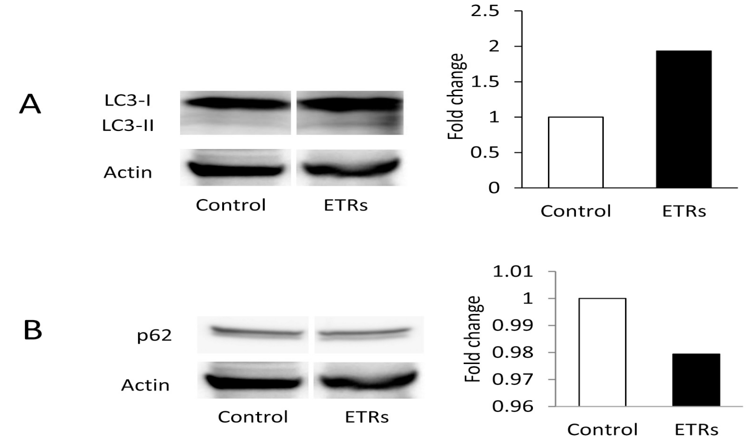

Supplementary figure 2

Figure S2. Western blotting of whole testicular tissue homogenate, showing upregulation of LC3-II and downregulation of p62 in ETRs, confirming light microscopic observations.

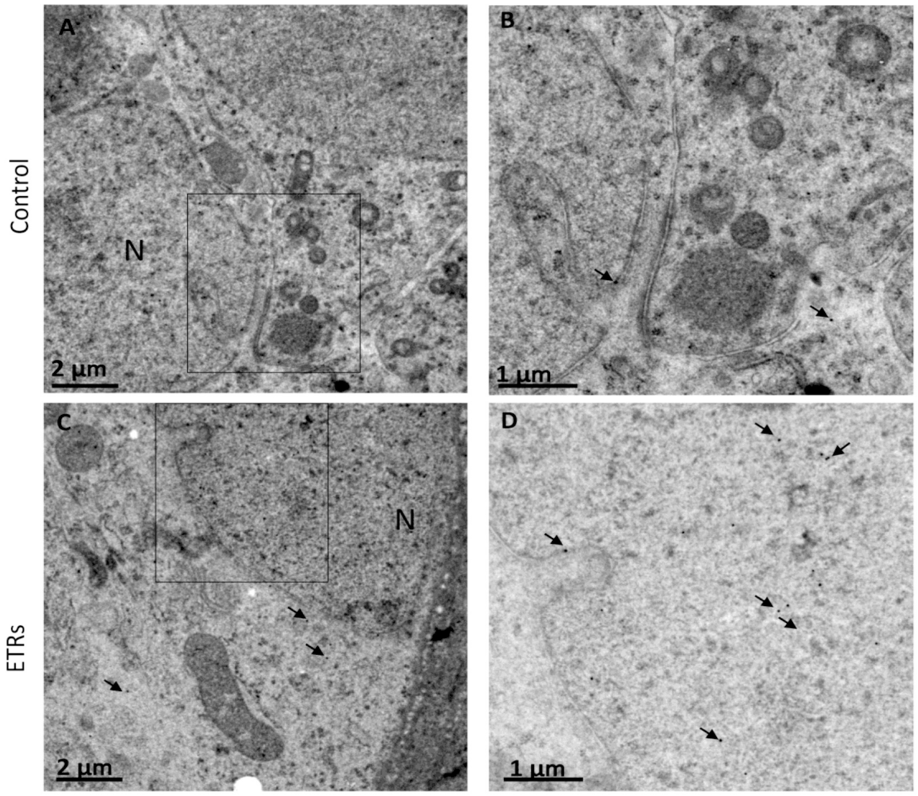

Supplementary figure 3

Figure S3. Immunogold labeling showing TFEB nuclear translocation in SCs of ETRs. The arrows indicate TFEB (15 nm) immunogold particles. N, SC nucleus. This method of post-embedding immunogold labeling is based on the manufacturer's protocols (Aurion, Wageningen, Netherlands; http://www.aurion.nl/products/gold_sols.php) and our recent publications [31,43].

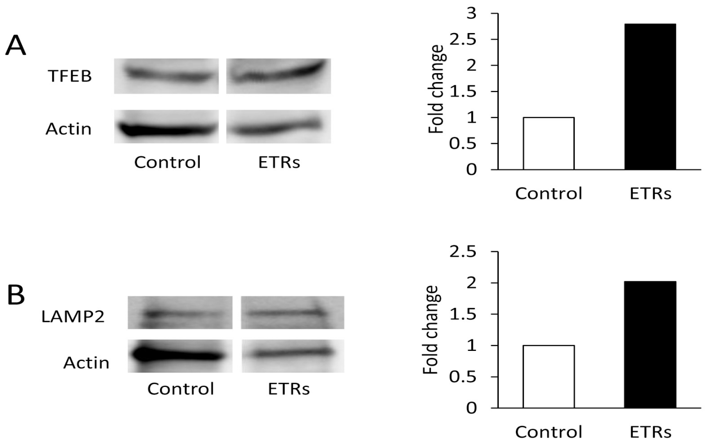

Supplementary figure 4

Figure S4. Western blotting of whole testicular tissue homogenate, showing upregulation of autophagy-related lysosomal proteins (TFEB and LAMP-2) in ETRs, confirming immunohistochemical study.

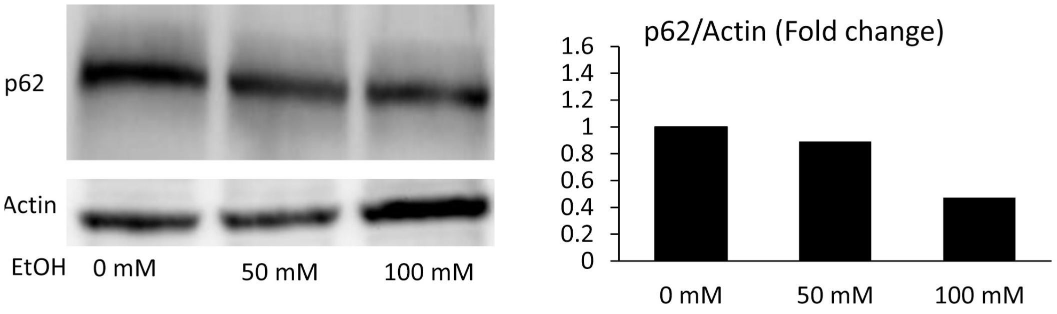

Figure S5. Downregulation of p62 in cultured ethanol-treated SCs.
